# Left Chylothorax Without Ascites in a Liver Transplant Patient

**DOI:** 10.7759/cureus.60996

**Published:** 2024-05-24

**Authors:** Benjamin J McCormick, Roy Archana

**Affiliations:** 1 Internal Medicine, Mayo Clinic, Jacksonville, USA

**Keywords:** ascites, chylous effusion, transjugular intrahepatic portosystemic shunt (tips, cirrhosis, chylothorax, liver transplant

## Abstract

Chylothorax is a rare condition that results from thoracic duct disruption with malignant and nonmalignant etiologies manifesting as a pleural effusion. Typically, chylothorax in the setting of cirrhosis is associated with the migration of chylous ascites. We present the case of a 64-year-old male with prior liver transplant who presented with new-onset transudative chylothorax without chylous ascites who responded to transjugular intrahepatic portosystemic shunt revision, diuresis, and serial thoracentesis.

## Introduction

Chylothorax is a rare but life-threatening condition due to chyle accumulation in the pleural cavity. Chyle consists of chylomicrons from the diet in the portal circulation, lower extremity lymphatic drainage, lymphocytes, and immunoglobulins with roughly 2.4 liters of production each day [1]. Known etiologies of chylothorax include thoracic duct disruption from malignancy, such as primary and metastatic lung cancers and lymphoma, radiation therapy, surgery, infections such as tuberculosis and histoplasmosis, yellow nail syndrome, Noonan syndrome, Cattleman disease, and autoimmune diseases [2]. Furthermore, chylous ascites with migration to the pleura can occur in patients with congestive heart failure and liver cirrhosis. Chylous ascites in cirrhotic patients most likely develops secondary to elevated portal pressures and diffuse degenerative changes in the lymphatic system leading to leakage of chyle into the peritoneal cavity [1,3-6]. The mechanism behind chylothorax formation in liver cirrhosis patients is unclear but is speculated to occur due to translocation of chylous ascitic fluid across small fenestrations in the diaphragm and negative intrapleural pressure [3-6]. We present the case of a rare left-sided chylothorax without ascites in a patient with prior liver transplantation and transjugular intrahepatic portosystemic shunt (TIPS). 

## Case presentation

A 64-year-old male with a past medical history of Crohn’s disease, chronic back pain, cirrhosis secondary to nonalcoholic steatohepatitis status post liver transplant (five years prior), and portal vein thrombosis on apixaban presented to the emergency department due to shortness of breath for two days. The patient had TIPS for recurrent ascites prior to transplantation and had a TIPS revision four years after transplantation to optimize his portosystemic gradient. His graft remained functional without evidence of rejection five years posttransplant. He denied chest pain, abdominal pain, fever, chills, productive cough, or unintentional weight loss. He denied sick contacts or recent travel. He denied any other symptoms above baseline. 

**Figure 1:**
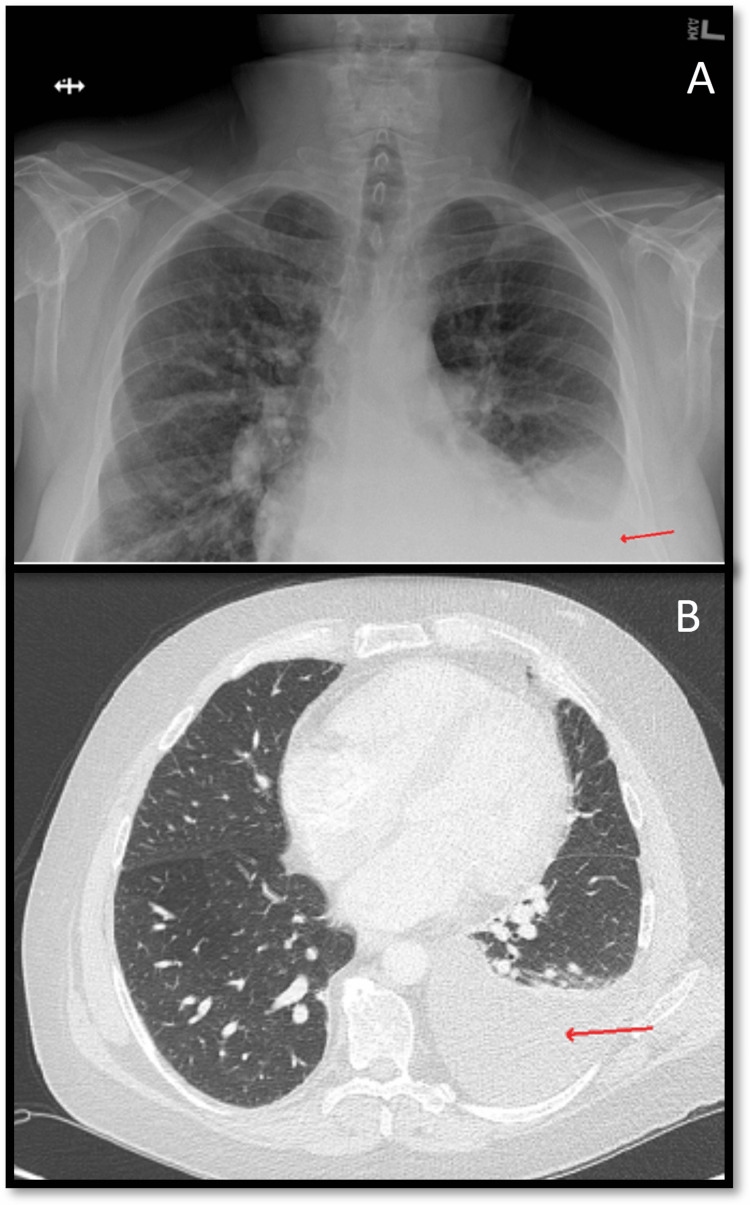
(A) Chest X-ray on admission demonstrates moderate left pleural effusion (arrow). (B) CT chest with IV contrast showing left-sided pleural effusion (arrow) one day after initial thoracentesis without evidence of mass effect or solitary nodules CT: Computed tomography; IV: intravenous

Vitals signs revealed a temperature of 36.8˚C, heart rate of 73 beats per minute, blood pressure of 129/73 mmHg, respiratory rate of 13 breaths per minute, and oxygen saturation of 93% on 3 liters of oxygen via nasal cannula. Physical examination revealed no peripheral edema and a nondistended, nontender abdomen. The patient exhibited nonlabored breathing with auscultation of the lungs revealing decreased air entry in the left base without rales, rhonchi, or wheezing. Abdominal ultrasound found insufficient ascites for paracentesis. Notably, a chest X-ray (CXR) one month prior to presentation revealed no pleural effusion. A CXR on admission showed a moderate left-sided pleural effusion (Figure 1A). A thoracentesis was performed with greater than 1 liter of orange, cloudy fluid drained. A subsequent CXR revealed near resolution of the pleural effusion with improvement in oxygen saturation greater than 90% on room air. The following day, the patient experienced recurrent hypoxia and dyspnea. A computed tomography (CT) chest with intravenous (IV) contrast showed re-accumulation of the left-sided pleural effusion without evidence of mass effect or solitary nodules (Figure 1B).

Pleural fluid studies showed a lactate dehydrogenase of 95 U/L (compared to serum lactate dehydrogenase 273 U/L), protein of 1.2 g/dL (compared to serum protein 6.4 g/dL), triglycerides of 164 mg/dL (compared to serum triglycerides 72 mg/dL), white blood cells of 636/µL with lymphocytic predominance, and cytology with reactive mesothelial cells without evidence of malignancy, which were consistent with a transudative effusion and the diagnosis of chylothorax. The patient was initiated on a low-fat diet.

On day three of hospitalization, the patient was evaluated by gastroenterology and interventional radiology who deemed that his TIPS was patent. However, the patient required daily thoracenteses due to recurrent effusions. On day four of hospitalization, he underwent a TIPS revision with resolution of dyspnea and slowed re-accumulation of the chylothorax, permitting discharge home with outpatient follow up. At a follow-up visit three months later, he had required thoracentesis only twice since the time of discharge. 

## Discussion

Chylothorax is a rare pathology due to thoracic duct disruption with clinical manifestations that can include malnutrition, respiratory failure, and death. One-third of chylothorax cases are left-sided, one-half are right-sided, and one-sixth of cases have bilateral involvement [2]. Chylothorax in the setting of portal hypertension is almost always associated with concomitant chylous ascites; however, there have been two reported cases, including our case, that transudative chylothorax may present without appreciable ascites [7]. 

The laterality of chylothorax is dependent on the site of the thoracic duct disruption. At the level of the fifth thoracic vertebra (T5), the thoracic duct inclines toward the left side to enter the superior mediastinum; thus, a disruption above T5 typically results in a left-sided chylothorax, whereas a disruption below T5 results in a right-sided chylothorax [8]. Diagnosis is confirmed by a pleural fluid triglyceride count greater than 110 mg/dL [1]. A lower triglyceride content can be seen in up to 15% patients in which case diagnosis can be confirmed with lipid electrophoresis [9]. 

Definitive treatments for chylothorax are primarily targeted at treating the underlying medical disease. However, symptomatic chylothorax should also be treated with a low-fat diet with increased medium chain triglycerides and a consideration of total parenteral nutrition (TPN) initiation given the high risk of malnutrition [1]. Furthermore, octreotide or other somatostatin analogues can be given to decrease portal hypertension. Thoracic duct ligation, pleurodesis, and pleurectomy are procedural interventions that can be utilized for chylothorax refractory to medical therapies [1]. Finally, TIPS is a safe and effective treatment for recurrent chylothorax in the setting of cirrhosis if there are no contraindications. The mechanism by which TIPS improves chylothorax remains speculative, although it is likely the result of reduced hepatic and gastrointestinal lymph flow via portal decompression [10]. Furthermore, this could explain why shunt dysfunction may result in recurrence of chylothorax, as seen in our patient. 

Although chylous ascites migration remains the primary postulated mechanism for chylothorax in patients with cirrhosis, our case reiterates the finding that transudative chylothorax may occur regardless of the presence of ascites in the setting of portal hypertension [7]. If the rate of ascites production equals the rate of chylothorax formation, a patient may present with chylothorax without chylous ascites.

## Conclusions

Liver cirrhosis is a rare cause of chylothorax. Chylothorax should be considered in patients with new-onset unilateral chylothorax in the setting of cirrhosis or prior liver transplantation. We present only the second reported case of isolated chylothorax related to cirrhosis without chylous ascites. Our case demonstrates that chylothorax may occur regardless of the presence of ascites in the setting of portal hypertension. Finally, TIPS is an effective treatment for patients with chylothorax due to underlying portal hypertension in patients with portal hypertension without ascites.
